# Predicting task performance in robot-assisted surgery using physiological stress and subjective workload: a case study with interpretable machine learning

**DOI:** 10.3389/fnhum.2025.1611524

**Published:** 2025-06-18

**Authors:** Kaiqi Wei, Chika Kimura, Megumi Shimura, Yoshihiro Shimomura, Xue Zhao, Takaaki Tamura, Shinichi Sakamoto

**Affiliations:** ^1^Division of Creative Engineering, Department of Design, Graduate School of Science and Engineering, Chiba University, Chiba, Japan; ^2^Design Research Institute, Chiba University, Chiba, Japan; ^3^Department of Urology, Graduate School of Medicine, Chiba University, Chiba, Japan

**Keywords:** robot-assisted surgery (RAS), neuroergonomics, physiological stress, surgeon workload, machine learning, SHAP (SHapley Additive exPlanations), task performance prediction

## Abstract

Robot-assisted surgery (RAS) enhances surgical precision and extends surgeons’ capabilities. However, its effects on the cognitive and physical states of surgeons remain poorly understood. It is essential to investigate the workload and physiological stress surgeons experience during RAS. This case study employs a neuroergonomic approach to explore how these factors relate to task performance. A single expert surgeon performed simulated surgical tasks under systematically varied conditions (noise level, surgical posture and task type) to elicit variations in stress and workload. During the tasks, multiple physiological signals were recorded, including electroencephalography (EEG), electromyography (EMG), heart rate (HR), and electrodermal activity (EDA). Subjective workload was also assessed using the NASA-TLX and SURG-TLX. Several classification models, including CatBoost, random forest, logistic regression, and support vector machines, were trained to predict task performance. Among them, CatBoost demonstrated the highest predictive accuracy (79.5%) and achieved an area under the curve (AUC) of 0.807. The model interpretation was conducted using SHapley Additive exPlanations (SHAP). The analysis revealed that subjective workload, mean HR, and muscle activation were the most influential predictors. EEG-related features contributed variably across conditions. This study shows that integrating subjective assessments with physiological measures can effectively predict surgical task performance under stress.

## 1 Introduction

Robot-assisted surgery (RAS) enhances instrument flexibility, stability, and surgical field visualization, helping to overcome the limitations of conventional laparoscopy (Chuchulo and Ali, 2023). However, it remains unclear how physiological stress and subjective workload during RAS relate to task performance under different stressors. Clarifying this relationship may enable the assessment, prediction, and improvement of surgical performance during RAS. Unlike conventional laparoscopy, RAS requires the surgeon to operate via a console. Although the console is ergonomically designed, prolonged operating in a static posture can lead to muscle fatigue and discomfort. As a result, RAS may simply shift postural stress rather than alleviate it (Catchpole et al., 2019). Additionally, the lack of non-verbal cues in communication with the surgical team may increase surgeons’ sensitivity to operating room (OR) noise, further elevating cognitive workload (Way et al., 2013; Tiferes et al., 2019). Both poor posture and high OR noise levels have been associated with increased workload and stress, potentially impair surgeons’ performance, raise the risk of errors, and compromise surgical safety (Arabacı and Önler, 2021; Li et al., 2023; Idrees et al., 2024).

The influence of intraoperative stressors on surgical performance has received growing attention. Researchers have investigated various indicators of stress and workload during surgery. Self-report scales remain a common method, but they are inherently subjective and may fail to capture real-time changes (Stefanidis et al., 2010; Mouraviev et al., 2016; Norasi et al., 2023; Fujiya et al., 2024). Wearable sensors provide an objective method to quantify stress and workload during surgery (Weenk et al., 2018; Morales et al., 2019; Pimentel et al., 2019; Yang et al., 2021; Shadpour et al., 2023). However, relying on a single physiological signal may not fully capture complex stress and workload responses. To address this, multimodal sensing, integrating electroencephalography (EEG), electromyography (EMG), electrocardiography (ECG), and electrodermal activity (EDA), has been proposed, offering a more comprehensive assessment (Zhou et al., 2020; Almukhtar et al., 2024). Although machine learning (ML) methods have been applied to surgical performance prediction, most existing studies focus on kinematic data and surgical video analysis to evaluate surgical skills (Zia et al., 2019; Nguyen et al., 2020; Schuler et al., 2023; Shafiei et al., 2023; Prevezanou et al., 2024). While previous studies have examined surgeon stress and workload during RAS, the predictive value of multimodal physiological signals and subjective assessments for task performance during RAS has not been systematically explored. This study aims to address this gap through a ML approach.

In this study, we conducted a controlled case study in which a single RAS surgeon performed simulated surgical tasks under varying conditions of task type, posture, and OR noise. To capture both objective and subjective indicators of stress and workload, we collected multimodal physiological signals—including EEG, EMG, heart rate (HR), and EDA—alongside self-reported workload assessments using NASA-TLX and SURG-TLX. ML models were then trained to predict task performance based on these data. To interpret the model outputs and identify key contributing features, we applied SHapley Additive exPlanations (SHAP) analysis. These findings may inform the development of real-time surgeon stress and workload monitoring, and performance optimization strategies in RAS.

## 2 Materials and methods

### 2.1 Participant

This case study involved an experienced RAS surgeon (> 250 RAS procedures). We aimed to explore the feasibility of subject-specific predictive modeling in assessing surgical performance. Focusing on one participant allowed us to examine intra-individual performance patterns. This design supports the development of accurate, personalized models and represents an initial step toward individualized surgical support systems. Prior to the study, the participant provided written informed consent and was explicitly informed that task difficulty and environmental stressors would be systematically manipulated as part of the experimental design.

### 2.2 Experimental setup

**FIGURE 1:**
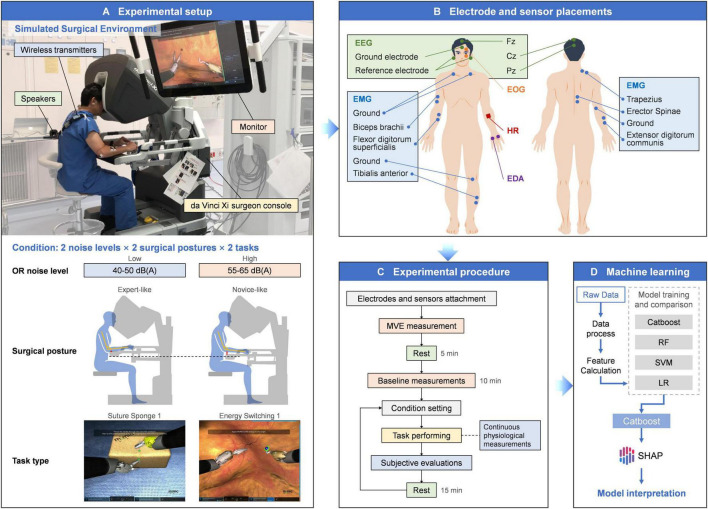
Comprehensive overview of the method. **(A)** Experimental setup. **(B)** Electrode and sensor placements for physiological measurements. **(C)** Experimental procedure. **(D)** Machine learning workflow.

The experimental setup is illustrated in Figure 1A. The participant performed two simulation tasks, Suture Sponge 1 (SS1) and Energy Switching 1 (ES1), using the da Vinci Skills Simulator (dVSS) connected to a da Vinci Xi surgeon console (Intuitive Surgical, Inc., Sunnyvale, CA, USA). Three independent variables, each with two levels, were applied to induce variability in stress and workload: OR noise level (low vs. high), surgical posture (expert-like vs. novice-like), and task type (SS1 vs. ES1).

High OR noise ranged from 55 to 65 dB(A), while low OR noise was set at 40–50 dB(A). Both conditions were played through speakers using a pre-recorded surgical environment soundtrack (Carillo et al., 2019). For surgical posture, the expert-like condition allowed the participant to adjust the console ergonomic controls for optimal comfort. In the novice-like condition, the armrest was set lower to reduce forearm support, replicating suboptimal ergonomics that may increase muscle tension (Franklin et al., 2003; Takayasu et al., 2018; Carillo et al., 2019).

The SS1 task involved manipulating a curved needle through two designated points on adjacent sponge faces using both hands. This task simulates essential suturing operations and demands a high degree of precision and focus. The ES1 task required the participant to adjust the camera view to identify and deal with multiple targets using appropriate instruments. It simulated multitask coordination in dynamic surgical scenario that requires rapid decision-making and frequent instrument switching. These two tasks were selected to represent distinct surgical skills. This approach helps reduce task-specific bias and improves the generalizability of the findings.

### 2.3 Data acquisition and feature calculation

This section describes the extracted physiological and subjective features. Physiological measures included EEG, EOG, EMG, EDA, and HR. Subjective workload was assessed using the NASA-TLX and SURG-TLX. All acquired data were preprocessed and analyzed to derive relevant metrics as features for subsequent modeling.

Physiological signals were continuously recorded throughout the experiment. The placement of electrodes and sensors for EEG, EOG, EMG, EDA, and HR measurements is illustrated in Figure 1B. EEG, EOG, EMG, and EDA signals were recorded at 1,000 Hz using the BIOPAC MP 160 system (BIOPAC Systems Inc., Goleta, CA, USA) with wireless transmitters and AcqKnowledge 5.0 (BIOPAC Systems Inc., Goleta, CA, USA). HR was measured with the Polar Verity Sense (Polar Electro Oy, Kempele, Finland) which uses photoplethysmography (PPG) and samples at 135 Hz for pulse detection. Subjective workload was assessed using paper-based versions of the NASA-TLX and SURG-TLX.

#### 2.3.1 EEG

Three scalp EEG electrodes were placed at the Fz, Cz, and Pz locations (10–20 system), selected as a minimal montage to reduce setup time and task interference while targeting regions associated with cognitive workload, motor control, and attentional processes. The recorded EEG data were processed in Acqknowledge 5.0 to remove EOG artifacts. In this study, the Beta-to-Alpha power ratio (BAR) was used as a stress metric (Roy et al., 2022). The powers in the α (8–13 Hz) and β (13–30 Hz) bands were computed by integrating the power spectral density (PSD), estimated using Welch’s method, over the corresponding frequency range using the trapezoidal rule. The BAR was then calculated. All computations were performed using Python.

#### 2.3.2 EMG

Surface EMG electrodes were attached to measure six muscle groups: the right upper trapezius, biceps brachii, extensor digitorum communis, flexor digitorum superficialis, erector spinae, and tibialis anterior. EMG signals were bandpass filtered from 20 to 450 Hz and notch filtered at 50 Hz to remove power-line interference (De Luca et al., 2010). The tibialis anterior was recorded from the left leg because in the experimental tasks, the participant operated only the camera and foot-clutch pedals on the left side, while the surgical instrument pedals near the right foot remained unused. This was due to the relatively basic nature of the tasks, which did not involve functions requiring right-foot pedal operation. Muscle activation was quantified as the percentage of maximal voluntary electrical activation (MVE%), calculated by dividing the root mean square (RMS) amplitude of the EMG signal during each task by the RMS amplitude obtained during a maximal voluntary contraction (MVC) (Dahlqvist et al., 2018).

#### 2.3.3 HR

The HR sensor was worn on the left forearm. Based on the recorded HR data, the mean HR and standard deviation of HR (SDHR), a first-order approximation of heart rate variability (HRV), were calculated (Levin and Swoap, 2019).

#### 2.3.4 EDA

The EDA electrodes were placed on the thenar and hypothenar eminences of the participant’s left hand. Recorded EDA data were processed in Acqknowledge 5.0, with a low-pass filter at 1 Hz, downsampled to 10 Hz, and then decomposed into phasic and tonic components through continuous decomposition analysis (CDA) in Ledalab V3.4.9 (Institute for Physiology, University of Graz, Austria). SCR were identified using a threshold of 0.01 μS. The number of significant SCR per second (nSCR/s) and the mean SCL were calculated (Lutnyk et al., 2023).

#### 2.3.5 NASA-TLX and SURG-TLX

The NASA-TLX measures subjective workload across six dimensions: mental demand, physical demand, temporal demand, performance, effort, and frustration (Hart and Staveland, 1988). The SURG-TLX is a surgical-specific scale developed based on the NASA-TLX. It includes six dimensions as well: mental demands, physical demands, temporal demands, task complexity, situational stress, and distractions (Wilson et al., 2011). The NASA-TLX employs a Visual Analog Scale (VAS) to measure each dimension, whereas SURG-TLX uses a 20-point Likert scale.

### 2.4 Experimental procedure

Figure 1C illustrates the Experimental Procedure. Two days prior to the experiment, the participant practiced both tasks to ensure familiarity and reduce performance variability. At the start of each session, the participant’s skin was cleaned, and conductive paste was applied before attaching the EEG electrodes. MVC was measured, followed by a five-minute rest period before a 10-min baseline recording of EEG, EDA, and HR.

The participant then performed the SS1 and ES1 tasks in a randomized order (three repetitions per task). Subjective evaluations were conducted after each task. A 15-min break was provided before the next trial to minimize fatigue and carryover effects. Over four consecutive days, the participant completed six unique task–posture–noise combinations (each with three trials) per day. Task order was counterbalanced to minimize order effects. All sessions started at consistent times to control for circadian variability. In total, the participant completed 72 trials (2 tasks × 2 noise levels × 2 postures × 9 repetitions), providing a comprehensive dataset for modeling.

### 2.5 Machine learning and SHAP analysis

Task performance was evaluated by dVSS as a composite score ranging from 0 to 100. This score incorporated completion time, master workspace range, and task-specific precision and error metrics (Havemann et al., 2019). Performance scores were dichotomized into high and low categories using a median split. The task performance prediction (high vs. low) was formulated as a supervised classification problem using the extracted features as input. Four classifiers were trained and compared: CatBoost, random forest (RF), support vector machine (SVM), and logistic regression (LR). CatBoost was chosen for its robustness in small datasets, leveraging ordered boosting to reduce overfitting, and its symmetric tree-growing strategy, which enhances training efficiency and model consistency. The model was trained with 355 trees and a learning rate of 0.015. These hyperparameters were manually selected to achieve a better balance between training and testing performance, rather than solely maximizing test AUC (Prokhorenkova et al., 2019). The RF model used 100 trees. The SVM model applied a radial basis function (RBF) kernel. The hyperparameters C and gamma were selected based on empirical performance across logarithmically spaced values. Logistic regression with L2 regularization was used as the baseline linear model. All models were implemented in Python. Figure 1D illustrates the overall workflow of the machine learning approach used in this study.

Model performance was evaluated using repeated 10-fold cross-validation. Performance metrics were averaged across folds to ensure reliability. We used the area under the curve (AUC) of the receiver operating characteristic (ROC) curve and accuracy, as well as additional confusion matrix metrics such as precision, recall, and F1-score to provide a comprehensive assessment.

After classification, SHAP was used to interpret the best model (Lundberg and Lee, 2017). Feature importance was quantified by averaging the absolute SHAP values across all trials. The distribution of SHAP values for each feature was further analyzed to evaluate its impact on the model’s predictions.

## 3 Results

### 3.1 Classification performance

**FIGURE 2:**
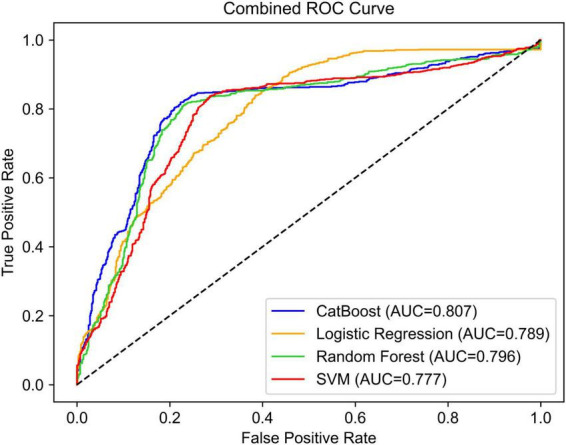
ROC curves for the four ML models to predict the participant’s simulated RAS task performance.

As shown in Figure 2, ROC curves were used to evaluate the performance of the four ML models. The CatBoost model achieved the highest test AUC of 0.807, outperforming random forest (0.796), logistic regression (0.789), and SVM (0.777), indicating its discriminative capability.

Additional performance metrics, including accuracy, precision, recall, and F1 score, are listed in Supplementary Table 1. CatBoost attained the highest accuracy of 0.795, along with similarly high precision (0.796), recall (0.793), and F1 score (0.795), suggesting its stable performance across multiple evaluation dimensions. Although random forest had the highest recall (0.818), its precision (0.770) and F1 score (0.793) were slightly lower than CatBoost. SVM showed moderately strong performance, particularly in recall (0.815), but had lower precision (0.749) and F1 score (0.781) compared to CatBoost and random forest. In contrast, logistic regression underperformed, especially in precision (0.696) and F1 score (0.726), indicating its limited effectiveness in this classification task. Overall, these results highlight CatBoost’s robustness across multiple evaluation metrics, supporting its role as the most effective model in this study.

### 3.2 Feature importance analysis

**FIGURE 3:**
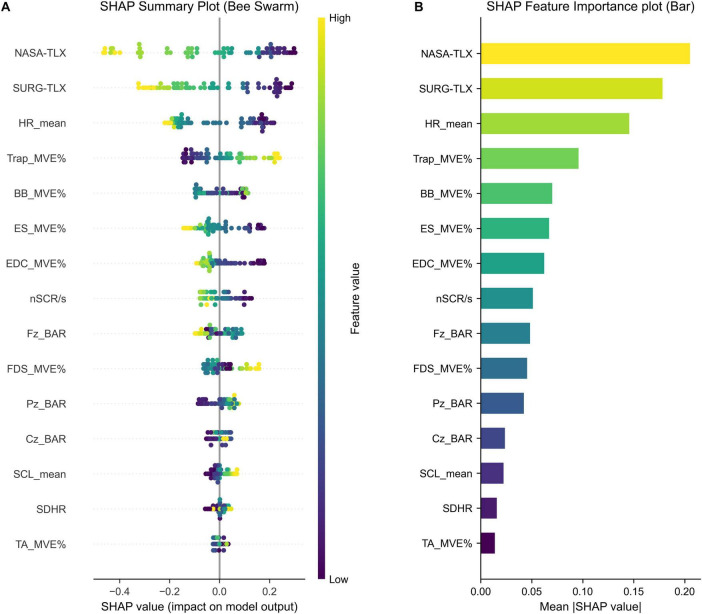
**(A)** SHAP summary plot (beeswarm) illustrating the distribution of SHAP values across all instances. Each dot represents a single trial, with color indicating the original feature value, ranging from high (yellow) to low (purple). Wider spread of dots along the *X*-axis indicates a greater impact on the model output. **(B)** SHAP feature importance plot (bar) showing the average magnitude of each feature’s contribution to the CatBoost model predictions. NASA-TLX, NASA task load index; SURG-TLX, surgery task load index; HR, heart rate; MVE%, percentage of maximal voluntary electrical activation; Trap, trapezius; BB, biceps brachii; ES, erector spinae; EDC, extensor digitorum communis; nSCR/s, number of skin conductance responses per second; BAR, Beta-to-Alpha power ratio; FDS, flexor digitorum superficialis; SCL, skin conductance level; SDHR, standard deviation of heart rate; TA, tibialis anterior.

As shown in Figure 3, the SHAP feature importance plot (bar chart) ranks input features based on their average absolute SHAP values, reflecting their overall contribution to the model’s predictions, while the SHAP summary plot (bee swarm) visualizes the distribution of SHAP values for each feature, capturing both the direction and magnitude of their effects on individual predictions. The NASA-TLX and SURG-TLX workload scores emerged as the top two most important predictors, suggesting a strong association between subjective workload assessments in predicting task performance. Additionally, the HR_mean and MVE% of several muscles (especially MVE%_Trap and BB_MVE%) were also identified as important features contributing to the model’s performance predictions. Conversely, Fz_BAR, Cz_BAR, and Pz_BAR exhibited relatively lower importance, suggesting that these EEG-derived features were less predictive of task performance in this context. Among EDA-related features, nSCR/s had moderate importance, while SCL_mean showed relatively low importance. Finally, SDHR and TA_MVE% demonstrated the lowest contribution to the model’s predictions. While the low importance of TA_MVE% reflects limited contribution from this specific muscle group, the result for SDHR should be interpreted with caution, as it is a simplified proxy that may not fully represent HRV.

## 4 Discussion

This study investigated the predictive value of physiological signals and subjective workload ratings for simulated RAS task performance under varying stress conditions. Using ML models and SHAP analysis, we examined the model-derived contributions of key features to performance predictions, highlighting the relevance of subjective workload measures and physiological metrics such as mean HR and trapezius MVE%. These findings offer insights into the selection into potential metrics for assessing workload and stress in RAS. It is important to emphasize that SHAP provides insights into model-derived associations rather than causal mechanisms. Therefore, all interpretations based on SHAP values should be regarded as correlational rather than causal.

To further clarify the contribution of this study, Table 1 compares it with recent work on surgical stress and performance. While prior studies often focused on a limited set of indicators, few have combined subjective workload measures, multimodal physiological signals, and interpretable machine learning methods. Our approach uniquely integrates SHAP-based model interpretation with validated workload scales and a comprehensive set of physiological signals.

**TABLE 1:** Predictive performances of the four ML models for predicting simulated RAS task performance.

References	Task type	Participants	Indicators	ML method	Objective	ML model interpretability
Lim et al., 2025	Real RAS	5 expert surgeons	EEG	None (statistical modeling)	Mental workload	N/A
Caballero et al., 2024	Real RAS	11 surgeons	EDA, blood pressure, body temperature, body movement	Multiple linear regression (MLR), SVM, multilayer perceptron (MLP)	Stress level	No
Zhou et al., 2020	Simulated RAS	12 surgeons	HRV, EDA, EEG	SVM, RF, k-nearest neighbors (KNN), LR	Mental workload	No
Takács et al., 2024	Simulated RAS (Sea Spikes)	5 medical students, 5 residents, 5 pre-cert surgeons	HRV, hand movement tracking, posture, SURG-TLX	Decision tree, KNN, SVM, LR	Skill level, stress level	No
This case study	Simulated RAS	1 expert surgeon	HR, EDA, EEG, EMG, NASA-TLX, SURG-TLX	SVM, LR, RF, CatBoost + SHAP	Surgical performance	Yes

### 4.1 Insights from psychophysiological indicators of workload and stress

The SHAP analysis indicated that NASA-TLX and SURG-TLX were among the most influential features in the model’s performance predictions, with higher workload scores associated with lower predicted performance in this modeling context. NASA-TLX appeared slightly more influential in the model’s output than SURG-TLX, which may reflect its broader sensitivity within this task context, though such interpretation should be drawn cautiously given SHAP rankings are model-specific. Given the simplified nature of the simulated RAS task, NASA-TLX may better capture workload variations. Some dimensions of SURG-TLX, such as task complexity and situational stress, may be less sensitive in this setting than in real surgical procedures. However, in real surgeries with greater procedural complexity and teamwork demands, SURG-TLX may demonstrate stronger predictive power (Ma et al., 2021). Nevertheless, since subjective evaluations cannot be conducted during actual surgeries, further research is needed to explore the relationship between subjective assessments and physiological indicators.

Among the autonomic nervous system metrics, mean HR exhibited the highest contribution to model predictions, consistent with prior research linking increased HR to higher cognitive workload and reduced performance (Mansikka et al., 2016; Sazuka et al., 2024). While the SHAP analysis showed a pattern where higher SDHR values tended to co-occur with better predicted performance, its overall contribution to the model output was relatively small. SDHR, calculated from PPG-derived HR rather than ECG-based R-R intervals, may lack robustness in reflecting autonomic modulation. Future studies should consider ECG to obtain more reliable HRV metrics such as RMSSD and HF/LF for a more accurate assessment of physiological stress and surgical performance (The et al., 2020).

nSCR/s ranked eighth in feature importance. Higher values were predominantly observed in low-performance trials. This finding supports the view that increased electrodermal activity reflects elevated physiological stress and cognitive load, which may interfere with motor execution. Excessive sympathetic activation has been associated with impaired motor control and cognitive overload during high-precision tasks (Awtry et al., 2025). In contrast, the mean SCL showed low feature importance and exhibited inconsistent patterns. This may be attributed to its sensitivity to chronic stress rather than acute task-related demands (Visnovcova et al., 2024). Further research is needed to clarify its relevance in predicting performance in real-world surgical environments.

The SHAP analysis revealed notable variability in the importance of MVE% across different muscles. The trapezius, biceps brachii, erector spinae, and extensor digitorum communis emerged as the most influential, while the flexor digitorum superficialis and tibialis anterior contributed less to model predictions. Higher MVE% in the erector spinae and extensor digitorum communis was associated with lower performance, which may reflect a pattern related to postural instability and increased hand exertion. Conversely, greater trapezius activation correlated with better performance, which may suggest an association with upper limb stabilization during surgical tasks (Rodrigues Armijo et al., 2020). Interestingly, the biceps brachii exhibited a U-shaped SHAP pattern. Both low and high activation levels were linked to improved performance predictions, suggesting that this muscle may activate differently depending on task demands. This pattern implies that distinct motor strategies may be associated with optimal performance under varying conditions (Takatoku and Fujiwara, 2010). It should be noted that SHAP provides model interpretability rather than causal inference. Therefore, further studies integrating EMG pattern analysis with kinematic data are required to clarify the functional contributions of these muscles.

BAR exhibited low overall predictive importance but displayed distinct SHAP patterns across the electrode sites. At Fz, a U-shaped relationship was observed, with extreme BAR values associated with poorer performance and moderate values linked to better outcomes. This trend aligns with the Yerkes–Dodson law, which suggests that moderate arousal may enhance performance, while excessive stress impairs it (Khazaei et al., 2021). At Cz, BAR did not demonstrate a clear pattern, possibly due to its primary role in motor control rather than cognitive workload (Shaw et al., 2019). At Pz, higher BAR correlated with improved performance, potentially indicating enhanced task monitoring or sensory integration under increased cognitive demands on Kahya et al. (2022). The use of only three EEG channels provided a practical, low-interference setup, but it limits the ability to analyze region-specific or lateralized brain dynamics involved in surgical tasks. Although BAR was not among the top predictors in this study, EEG remains a valuable modality for investigating neural mechanisms in surgical performance. Future research should explore additional EEG-derived metrics and refine task design to support more comprehensive analyses.

### 4.2 Practical implications and limitations

While several physiological indicators demonstrated predictive value, their application in real surgeries may be limited by practical constraints. For instance, certain EMG and EDA electrode placements may hinder precise surgical maneuvers, as noted by the participant. Although these measures offered valuable insights, future studies should carefully assess their feasibility in surgical environments. Our findings also highlight the potential of explainable ML in surgical performance assessment. SHAP analysis facilitates the identification of features that the model considered influential in its predictions, thereby enhancing interpretability. This kind of transparency is crucial for clinical adoption. If ML models can explain their feedback (e.g., showing that high EEG-based cognitive workload was associated with lower model-predicted performance), surgeons are more likely to trust and integrate them into surgical practice.

This study has several limitations. Most notably, it involved a single participant, which limits the generalizability of the findings. While the results offer initial insights, they cannot be extended to broader surgeon populations without replication in larger and more diverse samples. In particular, ML models trained on a single participant data may overfit to individual physiological or behavioral traits, limiting their applicability to other surgeons. Physiological indicators such as HR and EMG activation patterns can vary with fitness level, posture, and coping strategies. Additionally, subjective workload ratings like NASA-TLX may be influenced by individual interpretation bias. To minimize interference during task execution, we used a simplified EEG montage (Fz, Cz, Pz) and estimated HRV using PPG-derived HR instead of ECG. While both choices improved experimental feasibility, they limit the spatial resolution of EEG data and the robustness of autonomic stress measurements. Additionally, as SHAP values reflect the internal logic of a specific model, their interpretability is limited by model quality and data representativeness. Finally, our study involved a controlled environment, whereas real surgeries present dynamic stressors, such as time pressure, communication errors, or patient instability, which were not fully replicated.

Future research should compare stress-response patterns across surgeons with different experience levels, as key physiological indicators may vary (e.g., experts relying more on HRV, while novices exhibit elevated EDA associated with anxiety). Sensor design should also be optimized to minimize interference, exploring alternatives such as dry EEG electrodes integrated into surgical caps or other wearable devices. In addition, to obtain more precise autonomic stress analysis, future studies should use ECG rather than PPG, as ECG provides more reliable HRV metrics. Reducing the number of sensors to those with the highest predictive value, as identified in our SHAP analysis, could enhance the feasibility of real-time stress monitoring tools in surgical practice.

## 5 Conclusion

This brief report provides initial evidence that a combination of subjective workload assessments and physiological indicators, such as NASA-TLX scores, mean HR, and specific EMG-derived muscle activation, can predict surgical task performance in a simulated RAS environment. Among the machine learning models evaluated, CatBoost demonstrated the highest predictive accuracy (79.5%, AUC = 0.807), and SHAP analysis identified key physiological and cognitive features contributing to performance variability. These findings support the utility of explainable machine learning for uncovering interpretable relationships between stress, workload, and performance.

Although limited to a single participant and a controlled environment, this study highlights important indicators for real-time stress and workload monitoring in surgical contexts. Future work should validate these results across multiple surgeons and real surgical scenarios, while optimizing sensor configurations to minimize interference. Additionally, reducing sensor complexity based on SHAP-identified top contributors could improve feasibility in clinical settings. Research should also explore adaptive feedback systems or workload-aware robotic assistance to mitigate surgeon stress and enhance intraoperative performance and safety.

## Data Availability

The raw data supporting the conclusions of this article will be made available by the authors, without undue reservation.
