# Supplementary material for: Predicting task performance in robot-assisted surgery using physiological stress and subjective workload: a case study with interpretable machine learning
Source: Front Hum Neurosci. 2025 Jun 18;19:1611524. doi: 10.3389/fnhum.2025.1611524 (PMC12213715; doi:10.3389/fnhum.2025.1611524)
Supplement: Supplementary file 1 [file Table_1.docx]

**Table S1.** Predictive performances of the four ML models for predicting simulated RAS task performance

| **Machine learning model** | **AUC** | **Accuracy** | **Precision** | **Recall** | **F1 Score** |
| --- | --- | --- | --- | --- | --- |
| CatBoost | 0.807 | 0.795 | 0.796 | 0.793 | 0.795 |
| Logistic Regression | 0.789 | 0.714 | 0.696 | 0.760 | 0.726 |
| Random Forest | 0.796 | 0.787 | 0.770 | 0.818 | 0.793 |
| SVM | 0.777 | 0.771 | 0.749 | 0.815 | 0.781 |
